# Professional Etiquette*Paper presented at the Fourth Annual Meeting of the New York State Veterinary Medical Society.

**Published:** 1894-02

**Authors:** Claude D. Morris

**Affiliations:** Veterinary Surgeon


### PROFESSIONAL ETIQUETTE.*

By Claude D. Morris, Veterinary Surgeon.

In presenting a paper on the subject named, I must remind you that I find difficulties of no common character surrounding me at every point. Let me beg of you, therefore, to bear with me, should you consider my paper not sufficiently characteristic for so worthy a title. You may well ask, should not a gentleman of years of practice and extensive observation be better prepared to expound the principles which govern this subject, than one who has labored but a few years in so broad a field of usefulness?

However, feeling that I should not compromise with the subject or plead entire incapability, therefore we can speak in a certain degree from experience and observation.

In the struggle for maintenance and superiority in this life, it is as necessary that a professional man should possess tact and business sagacity, as it is that a ship should have a rudder. There are gentlemen in the ranks of our profession who are thoroughly acquainted with the scientific aspects of medicine; they can tell you in an alert way what to do for almost every disease incident to the lower animals; yet, after earnest endeavor, have failed to achieve reputation and acquire a practice commensurate to their ability, simply because they lack professional tact and business sagacity that would make their other qualities successful.

Again, there are men in the profession who are thoroughly qualified to practice and teach the tenets of the profession, but who are as thoroughly unscrupulous in their social and commercial relations as though they possessed no professional ability, and were but mere serfs. This latter class are the dangerous elements in the profession, possessing, as some of them do, alert and witty minds, with ready expressions to explain technical and scientific terms relating to medicine and treatment, readily comprehending and speaking with seeming knowledge the mysteries of pathology. This to a credulous world is looked upon as being smart; no doubt we can all of us call to mind living examples of these two classes that we have spoken of. These two classes can never be a success from a professional standpoint. The first lacks diplomacy, the other moral training, and in this day of enlightened civilization a man devoid of these elements, it were better had he not been born.

The corner-stone upon which to build a successful professional

*Paper presented at the Fourth Annual Meeting of the New York State Veterinary Medical Society.

life is manhood, the attributes of which are, the cultivation of whatever is true, whatever is just, and whatever is pure; found your expectations of success on these qualities and we will possess a personal and a scientific qualification. In the first place professional life is interwoven and environed with all classes of men; each profession has its sage and fool, its learned and highly cultured, its ignorant and rude. Classified as to vocation in the United States, the legal profession stand at the head, following close behind comes the medical; but when classified as to science, the medical is the guiding star of the century, outshining all others in the constellation of science. In the veterinary profession what course of action should a man pursue to bring him within the recognized meaning of professional etiquette? In the first place, what do we understand by the term Professional Etiquette? The word etiquette originally signified the giving of instructions on a card or piece of paper, prescribing the form of the ceremony at court or on other public occasions.

The word has come down to us to mean more than the order of ceremony. If we have authority by this time to prescribe set rules to govern professional conduct, the term is then elastic enough to prescribe for social behavior, and if for professional and social management, it then enters the domain of general instruction. If we accept this definition, we believe at the outset the location of one's office in the city or village is one of great importance. The practitioner should have his office on a street that is not objectionable to the best society; next door to a respectable place of business, with a pleasant front and a clean entrance, a neat, plain sign, Dr. William Jones, Veterinary Surgeon. The question of veterinary titles is one that must engage the attention of the profession at no distant day. It is unfortunate that the different institutions teaching the science, do not conform more in conferring of their degrees; it would be less confusing and more significant if they did. There are thirteen institutions in this country teaching veterinary medicine, either independently or in connection with a regular university course, and only four of this number agreeing as to title. The American nomenclature for veterinarians is very prolific in its store of titles, ranging as it does from a V.S., D.V.S., V.M.D., M.V.D., D.V.M. to an M.D.C. This array of titles is confusing to the public. What per cent. of the public, who need our services, would know what D.V.M. or M.D.C. was meant to convey unless told? The better part of society admires brevity, at least it should be comprehensive. You may think it a strained notion, but remember the appearance of one's office and its surroundings, compatible with the sense of an intelligent community, is the best introduction you could have to that community.

The subject of advertising is one that, as practitioners, we differ on. For my own part I believe that the least our names appear in the public print, from a professional standpoint, the more it will dignify the profession. After a prolonged absence from home, or recovery from sickness, it is wise and perfectly ethical to announce the fact of return to practice through the newspapers. Further than this I believe we should keep our names out of the newspapers and leave medical self-advertising to practitioners who prefer to do a quacking business.

I am convinced that a newspaper advertisement should consist simply of one's name as a veterinary surgeon, stating location of office, including office hours, and, if you like, further state that all calls will meet with prompt attention. Advertising the school from which we graduated, mentioning the honors received, etc., is superfluous, and is liable to be made an object of ridicule by those who desire to criticise. We find that a business or professional man is in closer touch with the needs of the community in which he is to discharge his duty, not to have a hobby save an aptitude for and diligent attention in the discharge of that duty, whatever it may be. I would not infer that we ought to become slaves to our profession or that we should couch in secret our opinions on the momentous questions of the day in relation to religion or politics. The individual who lacks the courage to own his God is not worthy of His beneficence. No sane man would demand of another a forbearance of his religious rights, and none but a political highwayman and a demagogue would dare to attempt to control the political faith of his neighbor.

Too many business and professional men are mute on these subjects, for fear they will lose a dollar in their business should it be known that their opinions were the opposite of that of their patrons on these questions. No, no, this cause is neither manly or ethical. Openly love your religion and embrace your political faith, and all right-thinking people will admire you. It is a mistaken notion that to be a successful veterinarian one must be able to fluently relate the characteristics, pedigree, and performances of trotting horses, from Lady Suffolk to Directum, or to be able to name the animal, under the same conditions, stating the merits, etc., of all the horses in their class, from a Pocahontas to Flying Jib or

Mascot. It certainly cannot enhance our knowledge in the treatment of disease to waste hours of study in tedious research to inform ourselves of the wonderful feats, from the time of Eclipse to that of Ormonde. This kind of information is valuable to a certain class of men, but is it practicable to the painstaking practitioner? The writer has been told by horsemen, that he would stand better as a veterinarian would he devote more time to the discussion of the above topic. My answer is, when I am called to treat a case of pneumonia, the paramount thought of my mind is to talk less and devote my energy to the successful accomplishment of that to which I was called. Neither do we believe that the public demands that we should be thoroughly posted on all the leading sports of the day. What is it to the profession whether Sullivan was ever pounded out by Corbett, or that Corbett and Mitchell will ever come to blows, and when they do, should they luckily kill each other, so much the better for the morals of the Commonwealth.

I believe one should be able to speak intelligently on the legitimate and better class of sports, but to make a hobby of any one of them, such would only betray to the public our weak points and indicate a corresponding inattention to the details of our profession.

We should be in possession of the merits of famous horses, and be able to speak of them with enthusiasm and correctness of detail in relation to them that would meet the approval of a conservative and intelligent community; but for veterinarians to sit for hours in the exclusive company of grooms and jockeys, indulging in periodical conversation and wagering nickels on the correctness of one another's statements in relation to the merits or demerits of horses of fame, is far from edifying, and is proof of the mental calibre of the participant.

It should be our ambition to be fairly well informed on the more important topics of the day, so as to be able, when in the company of intelligent gentlemen, to converse in a manner evincing an interest and to some degree an understanding of the great questions of our time.

Such discipline encourages a broad and tolerant conception of the motives and actions evolved in the minds of great men, whose energies move the world in its onward march to a higher civilization. It shoals the individual along the lines of a higher education; it makes him the companion of the moulders of great thoughts; it exemplifies him as a living pattern of the truth of his

Scriptural origin ; that he was made in the image of the Great Architect of the Universe, the ruler of the destinies of his people. Therefore we believe one of the imperative duties devolving upon professional life is that the individual should be alert with the times.

There is another feature in the discharge of our duties as veterinarians which would do much towards its elevation; it is to practice a closer relationship as professional brethren.

It should be the desire of the members of this Society to cultivate that spirit of respect for all members of the profession who are not of this Society, to the same degree as is entertained by those who are its members. A charitable recognition toward all reputable practitioners broadens the estimate and heightens the esteem of the community in behalf of the profession.

There are those of the profession who are not worthy of the regard of gentlemen. It would be a waste of energy and a compromise of one's self-respect to attempt an apology for the shortcomings and vice of these periodical nuisances, whose highest ambition is the company of the bar-room and leud associations; but on the other hand we should ever be ready to assist a brother practitioner in his times of misfortune, and especially in cases of probable litigation, should the plaintiff seek our opinions in the hope of securing substantial evidence to make his case appear stronger. Our first answer to such inquiry should be to discourage the intended action, on the grounds that the defendant is quite likely to produce expert testimony in support of his diagnosis and treatment, and that the unfavorable result, beyond all doubt, was only natural. I believe we should use all honorable means to destroy a case in court where an action has been brought against an honorable member of the profession, as in a great majority of these cases they are brought for gain and not for justice. A few successful actions against the veterinarians establishes a vicious custom in the community, as it places the practitioner face to face with unnecessary and unjust hazards, and encourages the stockowner to believe that he is entitled to indemnity in all cases where the results are unfavorable.

In short, do not allow legal precedents in these cases to be established, for should you be called before a court as the result of some unfortunate case, it would be much to your advantage to find that there was not a precedent in that particular case; in such an event the burden of evidence would rest largely upon the plaintiff.

I cannot advise too urgently the careful studying of the current literature, bearing directly upon the enlightenment of the science, published in this country. There are at present, so far as I know, three established veterinary periodicals, all published in the City of New York.

The JOURNAL OF COMPARATIVE MEDICINE AND VETERINARY ARCHIVES has reached its fifteenth year.

The *American Veterinary Review* has nearly completed its XVIIth vol., and the *New York Veterinary Journal and Record of Comparative Medicine,* a quarterly review, is well along in its IId vol.

It is needless for me to state that these journals are worthy the support of the profession. These periodicals are the result of much labor and expense on the part of those having them in charge. They are proof of the moral and intelligent support which these gentlemen are making in behalf of a higher status for the profession, and it is only our reasonable duty to give this laudable undertaking our co-operation. I believe this can best be accomplished by becoming their loyal patrons and by contributing a portion of our time in the careful preparation of some professional thesis on the more important cases coming under our care, setting forth in a clear, concise manner the best part of our professional activities. This would be beneficial in more ways than one. We believe the editors of these journals would give a welcome to such productions. We would not advise for a moment the communicating of an every-day practice to the sanctum of the editor. This would not only become burdensome to him, but would impose a much greater expense on the part of the management, and it would certainly fail of its intent; it would not be edifying. However, we are convinced a course of action such as has just been stated would be both dignified and productive of much good.

> '' Of all the arts in which the wise excel,
> Nature's chief masterpiece is writing well."

All people respect the man who thinks. It broadens one's field of thought and research, intensifies the desire for closer study of the literature of the profession, cultivates a quality admired of men, that of being able to contribute intelligently to the literature of one's profession. This sort of discipline creates a standing in professional circles, which extends beyond the limits of our practice, and gives to the practitioner a reputation which is quite as much to be desired as that of treating disease successfully.

The many details which compass this subject on every side are so numerous it becomes impossible to treat them, in a degree at al conclusive, in a paper like this. The features which demand consideration and have not been spoken of are quite as exigent as those that have been discussed.

In conclusion we will speak briefly and in general of principles of etiquette, which we should endeavor to emulate. They are found embodied in the code as prescribed by the Society. They are nothing more or less than a few business principles as a guide for professional conduct. We believe that they should be lived up to as closely as possible, as they possess the essence of our subject; but as we have intimated before, we believe that professional ethics cover a broader field and enter the domain of all the activities of our life. It does not mean simply the obedience of a few set rules; it is the exemplification in the daily walks of life with our fellow-men, the principles of moral philosophy.

To be ethical, as a professional man, signifies a character which is distinguished by strict honor, self-possession, forbearance, generous as well as refined feelings and polished deportment, a character to which all meanness and peevish fretfulness are alien, to which, consequently, a generous candor, scrupulous veracity, courage, both moral and physical, dignity, self-respect, and liberality in thought, argument, and conduct are habitual and have become natural.

The gentleman who is ethical in his professional conduct, possesses an equality of social claims and supercedes rank, office, or title. It passes the bounds of States and tongues; it gives him a passport acknowledged through the wide domain of civilization.

